# The association of quality of life and personality characteristics with adolescent metabolic syndrome: a cohort study

**DOI:** 10.1186/s12955-021-01797-7

**Published:** 2021-06-08

**Authors:** Xiaohua Liang, Peng Zhang, Shunqing Luo, Guifang Zhang, Xian Tang, Lingjuan Liu

**Affiliations:** 1 Clinical Epidemiology and Biostatistics Department, Children’s Hospital of Chongqing Medical University, Ministry of Education Key Laboratory of Child Development and Disorders, National Clinical Research Center for Child Health and Disorders, Key Laboratory of Pediatrics in Chongqing, China International Science and Technology Cooperation Center of Child Development and Critical Disorders, 136 2nd Street, Yuzhong District, Chongqing, 400016 China; 2 Disease Control and Prevention Center of Jiulongpo District, Chongqing, China; 3 Medical General Ward of Children’s Hospital of Chongqing Medical University, Chongqing, China; 4 Plastic Surgery Department of Children’s Hospital of Chongqing Medical University, Chongqing, China

**Keywords:** Quality of life, Personality traits, Adolescent, Metabolic syndrome, Cohort study

## Abstract

**Background:**

An increased prevalence of adolescent metabolic syndrome (MS) is associated with adulthood cardiovascular diseases. This study aimed to explore the potential relationship of quality of life (QoL) and personality traits with adolescent MS.

**Methods:**

A total of 1961 participants from Chongqing with an average age of 11.68 years old from a cohort study established in 2014 and followed up through 2019 were included. QoL information, Eysenck’s personality questionnaire and MS components were collected.

**Results:**

A higher QoL domain score of physical activity ability (PAA) was a protective factor for both MS and MS score (all *P* < 0.01), which was mainly negatively correlated with the MS components of central obesity, diastolic blood pressure (DBP) and triglyceride levels, as well as positively correlated with high density lipoprotein cholesterol (HDL-C) level. The total QoL score was negatively correlated with triglyceride levels and positively correlated with DBP (all *P* < 0.01). High extraversion personality score was a protective factor against adolescent MS (*P* = 0.04) and MS score (*P* < 0.05), which were mainly negatively correlated with the MS components of waist circumference, systolic blood pressure and TGs, and positively correlated with HDL-C (all *P* ≤ 0.01).

**Conclusions:**

QoL score and extraversion personality score were independent protective factors against both MS prevalence and MS score, suggesting that community intervention to improve the QoL and psychological health of children are essential.

**Supplementary Information:**

The online version contains supplementary material available at 10.1186/s12955-021-01797-7.

## Introduction

The increased prevalence of adolescent metabolic syndrome (MS) and MS component severity scores are independent predictors of adulthood cardiovascular diseases (CVDs) [1, 2]. MS components in adolescents include central obesity, elevated triglycerides (TGs), reduced high density lipoprotein cholesterol (HDL-C), elevated blood pressure and impaired fasting glucose. The prevalence of MS among adolescents ranged from 3.5 to 11.2% according to different regions and different diagnostic criteria [3, 4]. Current studies [5, 6] have found that shared genetic and environmental factors, family history of CVD and obesity, maternal gestational diabetes [7], low birth weight, early adiposity, social economic status (SES) [8], short duration of sleep [9], excessive screen time, dietary factors, low physical activity, and tobacco smoke exposure were potential risk factors for adolescent MS or MS components. The literature has consistently demonstrated evidence for the association of physical health risks and socioeconomic status with adolescent MS or its components; however, the association of health-related quality of life (QoL) and personality traits with adolescent MS should also gain the attention of researchers.

Limited studies [10] have illustrated the inverse relationship of QoL with MS or MS components mainly in adults, and the conclusions have been controversial. However, the majority of studies have shown that the association existed only in women [11] or existed only in subjects with depression [12]. Previous studies have revealed that the QoL domain scores of physical health [13] and social relationships [14], but not those of the mental health and perceived stress domains, were correlated with MS. Moreover, the impact factors of QoL were age, obesity, puberty development stage, SES, physical activity [15, 16] and unhealthy dietary behaviour [17], which were also correlated with MS. In addition, one community obesity intervention study [18] revealed that the control of obesity was associated with QoL improvement. Considered together, these studies suggest that QoL could have significant effects on MS, but evidence in children and adolescents has been scarce. Therefore, it is urgent to explore the association of QoL with adolescent MS and reveal the QoL domains that have salient effects.

Personality traits can impact MS and its components, although few studies have investigated this association. One study showed that children with obesity might experience several psychosocial problems [19]. A study found that extraverted personality is positively correlated with TGs, fasting blood-glucose (FBG) and MS scores in adults [20]. However, to our knowledge, there have been no studies exploring the correlations between personality traits and MS in adolescents, and personality traits tend to be stable over time and consistent across situations [21]. Therefore, personality traits could impact the prevalence of MS among children and adolescents, which must be illustrated.

In this study, we investigated whether QoL scores were inversely correlated with adolescent MS and MS components, which domains have salient effects, and whether the association was independent of age, sex, region and other variables. We investigated whether personality traits (extraversion, neuroticism, psychoticism) were associated with MS and MS components, and whether the association was independent of the influence of sex, age and other factors. A hypothesized framework figure is drawn in Additional file 1: Figure S1.

## Methods

### Subjects

**Fig. 1:**
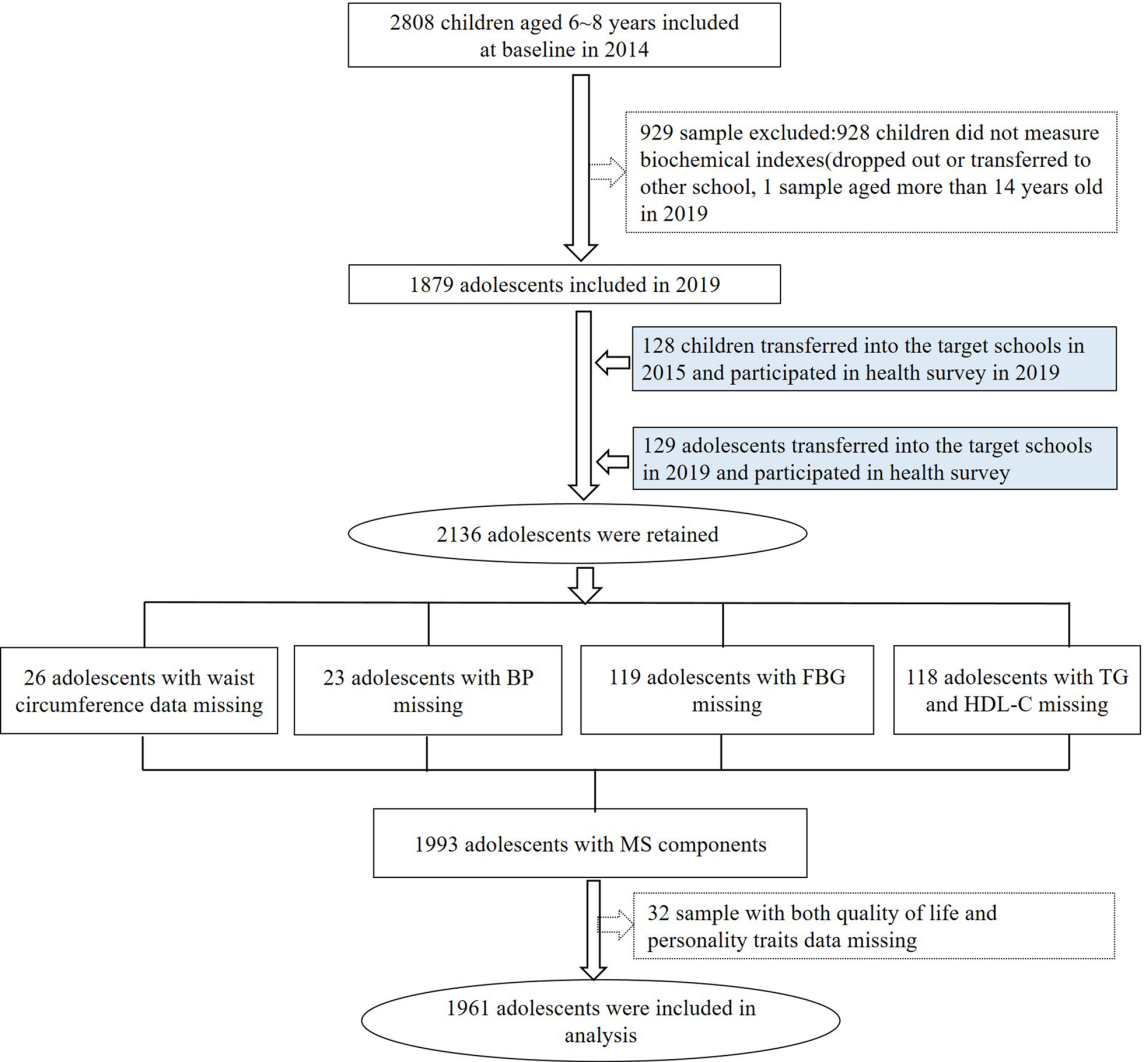
The flow chart of samples been included in the analyses

Two stage stratified cluster sampling was used to include participants from two counties in Chongqing that represent urban and rural areas; then, two regions per county were randomly selected, and 3067 children (including 2808 children who entered the cohort in 2014, and 130 and 129 children who transferred to the target schools in 2015 and 2019, respectively) were ultimately informed and included if they met the inclusion criteria. Participants who met all of the following criteria were recruited: (1) aged between 6 and 9 years old in 2014; (2) resided in the target region for more than 6 months; (3) did not have serious diseases (e.g., nephropathy, cardiovascular disease or cancer); and (4) had consent provided by the parents and children for participation. The sample size was calculated with the following parameters: α level of 0.05, power of 90%, prevalence of MS components of 15% and prevalence in the population of 10%, using the formula $n = p(1 - p)\left( {\frac{{z_{1 - \alpha /2} + z_{1} - \beta }}{{p - p_{0} }}} \right)^{2}$. Assuming an attrition rate of 20%, 1859 participants were needed, and 1961 subjects were ultimately included in this study (as shown in Fig. 1). The general characteristics of the included and excluded samples are described in Additional file 2: Table S1. At baseline, all of the participants completed the SES and family health history questionnaires and were recruited mainly from grade one and grade two based on primary public school screening of children whose families were interested in health research. The questionnaires were administered and collected by the teachers. The Institutional Review Board at the Children’s Hospital of Chongqing Medical University provided approval for the study with an ethics approval number (2019-86). Informed consent was provided by all of the subjects and parents/guardians.

### Demographic variables

Demographic information, SES and prenatal variables were collected. The validity and reliability of the demographic questionnaire were checked and are described in detail in a previous publication [22]. The demographic questionnaire was completed by the parents or guardians of the children after standard training by the research group, and detailed instructions regarding the questionnaire were given to the parents or guardians.

### Physical examination

Anthropometric measurements were conducted both in 2014 and in 2019 by well-trained paediatric nurses, and the protocol for these measurements was described in a previous publication [22]. Waist circumference (WC) was used as an alternative measure of central adiposity. Hip circumference was measured twice horizontally at the level of the pubic symphysis in the front and the gluteus maximus in the back, with the participant standing upright and with his or her legs together and placing the arms naturally at the sides; the mean value was used.

Blood pressure (BP) was measured on three separate occasions with an OMRON arm-type electronic sphygmomanometer (HEM7051) using an appropriately sized BP cuff placed on the subject’s right arm and with the subject in a seated position, as described in detail in a previous publication [22].

### Biochemical indexes

The biochemical markers FBG, HDL-C, TG, low density lipoprotein cholesterol (LDL), and total cholesterol (TC) were measured in 2014 and 2019. Venous blood (3 mL) was drawn from each subject in the morning after at least 12 h of fasting and 24 h of abstaining from high-fat and spicy foods. The biochemical markers were measured within 2 h after venous blood was drawn using the protocol introduced in detail in our previous publication [22].

### Measurement of QoL and Eysenck’s Personality Questionnaire (EPQ)

The QoL questionnaire for adolescents consists of 49 items, including 4 factors (psychosocial function, physical and mental health, living environment and QoL satisfaction) and 13 dimensions, including self-satisfaction, relationship of teacher and pupil, physical feeling, companionship, parenthood, physical activity ability (PAA), learning ability and attitude, self-esteem, negative emotion, attitude towards completing homework, opportunity for activity, living convenience and others (picky-eating and surroundings), as detailed in Additional file 3: Figure S2. The order of presentation of the 49 items was randomized. Children rated the statements on a 4-point scale, and the direction of response (positive or negative) varied item by item to limit response bias. Individual item values were recoded prior to analysis such that the direction was consistent. Responses were totalled and normalized according to the age-, sex- and region-specific norms of China into a score T (range 0–100), using the function T = 50 + (X-M)/SD × 10, with higher scores suggesting a better QoL status [23].

The Chinese version of the Eysenck personality questionnaire[24] consisted of 88 items scored on a 2-point scale (for positive items NO = 0 and YES = 1), including 4 domains: extraversion (E) (25 items), neuroticism (N) (23 items), psychoticism (P) (18 items), and lie scales (L) (22 items). High scorers on the E scale indicate sociable, exciting, pleasurable, carefree, and aggressive characteristics. A higher score on the N scale is more likely to indicate a worried and moody person who tends to suffer from emotional and psychosomatic disorders. The P scale was designed to measure behaviour patterns that could be considered schizoid or psychopathic in extreme cases. The L scale assesses response bias. Items in the E, N, P and L domains are totalled and normalized to the age- and sex-specific norms into a score ranging from 0 to 100 using the function T = 50 + (X-M)/SD × 10. People were defined as middle type, tendency type and typical type based on T score (E and N) ranges of 43.3–56.7, 38.5–43.3 or 56.7–61.5, and < 38.5 or > 61.5, respectively. People were considered to have a psychotic personality if the T score of the P domain was > 56.7. We considered the responses regarding personality traits invalid if the T score of the L domain was > 70.

Fifty samples were required to complete the same questionnaire twice within a one-week interval to check the validity and reliability of the QoL and Eysenck’s Personality Questionnaires before our formal survey. QoL and Eysenck’s Personality Questionnaires were completed by the adolescents after a standard training.

### Diagnostic criteria

MS in adolescents was defined by the presence of three or more of the following five components [5, 25]: (1) central obesity defined as ≥ 90th percentile for age and gender criteria in China [26]; (2) elevated systolic and/or diastolic blood pressure ≥ 90th percentile for age, sex and height (according to the study by Jie Mi [27]); (3) hypertriglyceridaemia defined as TG ≥ 1.24 mmol/L; (4) low serum HDL-C defined as HDL-C ≤ 1.03 mmol/L, and (5) impaired fasting glucose (IFG) defined as FBG ≥ 5.6 mmol/L. Next, individual MS scores were calculated as the sum of the number of MS components present (range 0–5).

The definitions of size for gestational age were based on the global reference for foetal-weight and birthweight percentiles [28]: birth weight at or greater than the 90th percentile indicated large for gestational age (LGA), and birth weight less than the 10th percentile indicated small for gestational age (SGA), using the parameters of mean birthweight at 40.5 weeks of 3332.93 g and a variation coefficient of 14.36%.

### Statistical analyses

Differences in anthropometric measures, serum biochemical indexes, QoL and personality score among the three groups were assessed using ANOVA, and post hoc comparisons were performed using the Student–Newman–Keuls (SNK) test. Type I errors were already adjusted. We selected potential covariates if the difference was significant (*P* < 0.05), and compared the differences among MS component groups (No MS Components, 1–2 MS Components and MS). The χ^2^ test was used to test the difference in the component ratio of potential risk factors for MS components. A logistic regression model was performed using diagnosed MS components or MS as the dependent variables with QoL and personality traits, as independent variables, and adjusted for covariables. In addition, a generalized linear model (GLM) was used to analyse the correlation of QoL and personality trait scores with MS component levels and MS scores and adjusted covariables.

The data analysis was conducted using SAS software, version 9.4 (Copyright © 2020 SAS Institute Inc. Cary, NC, USA). A significant difference was defined by an α level of 0.05.

## Results

### General characteristics

The general characteristics of the subjects are shown in Table 1. A total of 1961 samples were included, with a follow-up rate of 63.94% (1961/3067). The mean age was 11.68 ± 0.60 years old (7.26 ± 0.59 years at baseline), and 52.01% (1020/1961) were male. The prevalence of adolescent MS was 4.69% (92/1961), and the prevalence rates of MS components were 21.83% (428/1961), 25.45% (499/1961), 9.28% (182/1961), 9.79% (192/1961) and 1.53% (30/1961) for central obesity, elevated TGs, reduced HDL-C, elevated BP, and IFG, respectively. Anthropometric measures, biochemical indexes, MS components, perinatal, and SES variables, QoL score, and personality characteristics among the MS groups are shown in Table 1. Age, region, foetal weight of pregnancy week and mother’s education level, which were adjusted in the multivariable analysis, showed significant differences among the three groups (no MS component, 1–2 MS components and MS) (all *P* < 0.05).

**Table 1:** General characteristics of adolescent QoL and personality traits with MS study

Variables	All	Metabolic syndrome (MS)			
		No	1 ~ 2 MS Components	Yes	*P*
Sample size	1961	1052 (53.65%)	817 (41.66%)	92 (4.69%)	
**Region**					
Urban	1467 (74.81%)	829 (78.80%)	576 (70.50%)	62 (67.39%)	< 0.01
Rural	494 (25.19%)	223 (21.20%)	241 (29.50%)	30 (32.61%)	
Gender, male (n (%))	1020 (52.01%)	554 (52.66%)	415 (50.8%)	51 (55.43%)	0.58
Age, y	11.68 ± 0.60	11.65 ± 0.60	11.72 ± 0.60	11.75 ± 0.60	0.04
**Anthropometric measures in 2014**					
Age, y	7.26 ± 0.59	7.24 ± 0.59	7.28 ± 0.58	7.29 ± 0.60	0.33
Height, cm	124.52 ± 6.11	123.67 ± 5.86_a_	125.27 ± 6.15 _b_	127.57 ± 6.72 _c_	< 0.01
Weight, kg	25.92 ± 5.58	24.27 ± 4.13_a_	27.27 ± 5.98 _b_	32.65 ± 7.61 _c_	< 0.01
BMI, kg/m2	16.59 ± 2.53	15.79 ± 1.82_a_	17.24 ± 2.75 _b_	19.84 ± 3.09 _c_	< 0.01
Waist circumference, cm	55.69 ± 7.09	53.59 ± 5.55_a_	57.40 ± 7.48 _b_	64.23 ± 8.46 _c_	< 0.01
SBP, mmHg	100.6 ± 8.94	98.85 ± 8.36_a_	102.14 ± 9.04 _b_	106.70 ± 9.33 _c_	< 0.01
DBP, mmHg	62.68 ± 7.76	61.57 ± 7.51_a_	63.72 ± 7.79 _b_	66.08 ± 8.04 _c_	< 0.01
**Anthropometric measures in 2019**					
Height, cm	151.75 ± 7.98	150.22 ± 7.89_a_	153.21 ± 7.67_b_	156.33 ± 7.59_c_	< 0.01
Weight, kg	44.30 ± 10.98	39.63 ± 7.69_a_	48.58 ± 11.24_b_	59.62 ± 11.30_c_	< 0.01
BMI, kg/m^2^	19.07 ± 3.76	17.45 ± 2.50_a_	20.58 ± 4.01_b_	24.22 ± 3.32_c_	< 0.01
Waist circumference, cm	65.83 ± 10.03	61.20 ± 5.98_a_	70.06 ± 10.77_b_	81.18 ± 8.26_c_	< 0.01
Hip circumference, cm	81.68 ± 8.28	78.45 ± 6.49_a_	84.68 ± 8.41_b_	92.05 ± 6.58_c_	< 0.01
SBP, mmHg	105.71 ± 9.49	102.76 ± 8.02_a_	108.29 ± 9.62_b_	116.62 ± 9.36_c_	< 0.01
DBP, mmHg	62.82 ± 6.70	61.40 ± 5.87_a_	63.98 ± 7.07_b_	68.72 ± 7.05_c_	< 0.01
Puberty	536 (27.33%)	267 (25.38%)	246 (30.11%)	23 (25.00%)	0.11
***Serum biochemical indexes in 2014***					
FBG, mmol/l	4.12 ± 0.6	4.07 ± 0.59_a_	4.17 ± 0.58_ab_	4.29 ± 0.71_b_	< 0.01
TC, mmol/l	3.51 ± 0.72	3.49 ± 0.75	3.54 ± 0.68	3.62 ± 0.69	0.25
TG, mmol/l	0.91 ± 0.53	0.82 ± 0.42_a_	0.99 ± 0.58_b_	1.36 ± 0.78_c_	< 0.01
HDL-C, mmol/l	1.26 ± 0.27	1.30 ± 0.27_a_	1.23 ± 0.27_b_	1.10 ± 0.22_c_	< 0.01
LDL-C, mmol/l	1.73 ± 0.56	1.68 ± 0.53_a_	1.78 ± 0.59_a_	1.99 ± 0.63_b_	< 0.01
***Serum biochemical indexes in 2019***					
FBG, mmol/l	4.45 ± 0.43	4.45 ± 0.38	4.45 ± 0.49	4.50 ± 0.51	0.58
TC, mmol/l	3.52 ± 0.61	3.53 ± 0.57	3.50 ± 0.66	3.47 ± 0.62	0.43
TG, mmol/l	1.06 ± 0.50	0.83 ± 0.20_a_	1.26 ± 0.55_b_	1.85 ± 0.84_c_	< 0.01
HDL-C, mmol/l	1.44 ± 0.31	1.55 ± 0.28_a_	1.34 ± 0.28_b_	1.06 ± 0.20_c_	< 0.01
LDL-C, mmol/l	1.84 ± 0.44	1.81 ± 0.42_a_	1.86 ± 0.46_a_	1.94 ± 0.46_b_	< 0.01
***Metabolic syndrome components***					
Central obesity	428 (21.83%)	_	341 (41.74%)	87 (94.57%)	< 0.01
Elevated triglycerides	499 (25.45%)	_	416 (50.92%)	83 (90.22%)	< 0.01
Reduced HDL-C	182 (9.28%)	_	122 (14.93%)	60 (65.22%)	< 0.01
Elevated blood pressure	192 (9.79%)	_	141 (17.26%)	51 (55.43%)	< 0.01
Impaired fasting glucose	30 (1.53%)	_	27 (3.30%)	3 (3.26%)	< 0.01
***Perinatal measures***					
**Fatal weight of pregnancy week**					
Appropriate for gestational age	1097 (68.18%)	599 (69.41%)	444 (66.37%)	54 (70.13%)	0.04
Small for gestational age	126 (7.83%)	79 (9.15%)	43 (6.43%)	4 (5.19%)	
Large for gestational age	386 (23.99%)	185 (21.44%)	182 (27.20%)	19 (24.68%)	
***Mother’s education, y***					
~ 9	641 (33.42%)	335 (32.56%)	283 (35.42%)	23 (25.56%)	0.04
~ 12	660 (34.41%)	363 (35.28%)	254 (31.79%)	43 (47.78%)	
≥ 15	617 (32.17%)	331 (32.17%)	262 (32.79%)	24 (26.67%)	
***13 domains of QoL***					
Self-satisfy	50.07 ± 11.35	49.81 ± 11.32	50.54 ± 11.40	48.99 ± 11.18	0.25
Relationship of teacher and pupil	53.59 ± 10.08	53.50 ± 9.97	53.82 ± 10.09	52.67 ± 11.29	0.53
Physical feeling	49.94 ± 10.75	49.97 ± 10.57	49.99 ± 10.89	49.25 ± 11.61	0.82
Companionship	53.74 ± 10.51	53.42 ± 10.46	54.33 ± 10.30	52.24 ± 12.49	0.07
Parenthood	51.27 ± 11.36	51.18 ± 11.17	51.45 ± 11.70	50.81 ± 10.64	0.82
Physical activity ability	50.14 ± 10.66	50.93 ± 10.45_a_	49.48 ± 10.80_a_	46.89 ± 10.84_b_	< 0.01
Learning ability and attitude	51.77 ± 10.24	51.50 ± 10.15	52.26 ± 10.16	50.53 ± 11.69	0.14
Self-esteem	50.34 ± 11.06	50.17 ± 10.96	50.76 ± 11.09	48.64 ± 11.83	0.17
Negative emotion	47.71 ± 11.25	47.57 ± 11.26	48.05 ± 11.09	46.41 ± 12.47	0.34
Attitude of doing homework	51.47 ± 9.06	51.57 ± 8.92	51.48 ± 9.05	50.25 ± 10.77	0.41
Activity opportunity	54.60 ± 9.62	54.16 ± 9.59	55.26 ± 9.64	53.69 ± 9.46	0.03
Living convenience	54.45 ± 7.73	54.28 ± 7.60	54.57 ± 7.96	55.18 ± 7.18	0.47
Other (picky-eating and surroundings)	50.66 ± 10.12	50.04 ± 10.24_a_	51.25 ± 9.93_a_	52.49 ± 10.03_b_	< 0.01
***Four factors of QoL***					
Psychosocial health factor	64.79 ± 10.37	64.64 ± 10.19	65.18 ± 10.46	63.13 ± 11.45	0.46
Physical and mental health factor	35.87 ± 5.98	35.89 ± 5.87	35.94 ± 6.06	35.12 ± 6.50	0.16
Living environment factor	24.01 ± 4.28	24.14 ± 4.24_a_	23.94 ± 4.33_a_	23.11 ± 4.19_b_	0.07
Quality of life satisfaction factor	24.90 ± 4.37	24.75 ± 4.40	25.11 ± 4.36	24.79 ± 4.21	0.21
Total score of QoL	52.61 ± 12.37	52.47 ± 12.17_ab_	53.06 ± 12.56_a_	50.33 ± 12.85_b_	0.12
***Personality characteristics***					
Extraversion (E)	50.30 ± 13.04	50.69 ± 13.19_a_	50.13 ± 12.86_a_	47.33 ± 12.67_b_	0.05
Neuroticism (N)	50.28 ± 14.67	50.48 ± 14.55	49.80 ± 14.60	52.31 ± 16.59	0.26
Psychoticism (P)	39.86 ± 8.96	39.92 ± 8.94	39.71 ± 8.86	40.58 ± 10.11	0.66
Lie (L)	53.43 ± 8.89	53.41 ± 8.67	53.41 ± 9.09	53.89 ± 9.68	0.88

a,b,c: the difference among groups using “a”, “b”, “c” labelled, different letters mean the difference existed between two groups

Table 1 displays the QoL and personality trait scores of children with different MS component scores. Adolescents with MS had lower QoL domain scores for PAA, activity opportunities and other (picky eating and surroundings) than their counterparts with no or 1–2 MS components (*P* < 0.01, *P* = 0.03 and *P* < 0.01). In addition, the extraversion score of personality traits was decreased in children with MS compared with their counterparts with no MS components or 1–2 MS components (*P* = 0.05).

### Relationship of elevated MS components with QoL and personality scores

The association of elevated MS components in 2019 with adolescent QoL and personality traits in 2019 is shown in Additional file 4: Table S2 (unadjusted covariates) and in Table 2 (adjusted covariates). After adjusting for covariates, an elevated domain scores of PAA (OR (95% CI) 0.964 (0.953, 0.975), *P* < 0.01) and living environment factor scores (OR (95% CI) 0.948 (0.921, 0.977), *P* < 0.01) were protective factors against central obesity. However, the domain score of others (picky-eating and surroundings) was positively correlated with central obesity (OR (95% CI) 1.014 (1.001, 1.026), *P* = 0.03), elevated BP (OR (95% CI) 1.027 (1.009, 1.045), *P* < 0.01) and decreased HDL (OR (95% CI) 1.024 (1.006, 1.042), *P* = 0.01). The QoL domain scores of self-satisfaction, physical feeling, negative emotion and factor scores of physical and mental health and quality of life satisfaction were positively correlated with elevated BP (in Table 2). In contrast, personality trait scores of extraversion (OR (95% CI) 0.985 (0.972, 0.998), *P* = 0.02) were negatively correlated with elevated BP. In addition, an elevated domain scores of QoL of self-satisfaction, physical feeling, parenthood, PAA, negative emotion, factor score of physical and mental health and total score of QoL were correlated with elevated TGs, while neuroticism score was positively correlated with elevated TGs (in Table 2). The domain scores of physical feeling (OR (95% CI) 1.049 (1.002, 1.099), *P* = 0.04) and activity opportunity (OR (95% CI) 1.054 (1.005, 1.107), *P* = 0.03) were positively correlated with IFG.

**Table 2:** Logistic regression analysis of the association of QoL and personality traits with adolescent MS components

Variables	Central obesity		Elevated BP		Elevated TGs		Decreased HDL		Impaired fasting glucose	
	OR (95%CI)	*P*	OR (95%CI)	*P*	OR (95%CI)	*P*	OR (95%CI)	*P*	OR (95%CI)	*P*
***Part 1:Relationship of QoL and personality scores with elevated MS components in 2019***^**a**^										
**Domains of QoL**										
Self-satisfaction	0.997 (0.986, 1.007)	0.56	1.018 (1.002, 1.036)	0.03	0.989 (0.98, 0.999)	0.04	1.004 (0.989, 1.019)	0.60	1.024 (0.983, 1.065)	0.26
Physical feeling	0.995 (0.984, 1.006)	0.38	1.019 (1.002, 1.037)	0.03	0.989 (0.979, 1)	0.04	0.998 (0.983, 1.014)	0.80	1.049 (1.002, 1.099)	0.04
Parenthood	0.998 (0.987, 1.009)	0.73	1.016 (0.999, 1.033)	0.07	0.99 (0.98, 1)	0.05	1.006 (0.991, 1.022)	0.45	1.005 (0.967, 1.043)	0.81
Physical activity ability	0.964 (0.953, 0.975)	< 0.01	0.998 (0.982, 1.014)	0.79	0.983 (0.972, 0.994)	< 0.01	1 (0.984, 1.015)	0.96	1.022 (0.981, 1.064)	0.30
Negative emotion	1.001 (0.991, 1.012)	0.80	1.017 (1.001, 1.033)	0.04	0.989 (0.979, 0.999)	0.03	1.003 (0.988, 1.018)	0.71	1.026 (0.988, 1.066)	0.18
Activity opportunity	0.998 (0.985, 1.011)	0.74	1.015 (0.997, 1.034)	0.10	1.002 (0.989, 1.014)	0.80	0.993 (0.976, 1.011)	0.46	1.054 (1.005, 1.107)	0.03
Other	1.014 (1.001, 1.026)	0.03	1.027 (1.009, 1.045)	< 0.01	1.001 (0.99, 1.013)	0.81	1.024 (1.006, 1.042)	0.01	1.024 (0.982, 1.068)	0.27
**Four factors of QoL**										
Physical and mental health	0.994 (0.975, 1.015)	0.59	1.037 (1.006, 1.07)	0.02	0.976 (0.958, 0.995)	0.02	1 (0.972, 1.029)	0.99	1.056 (0.98, 1.139)	0.15
Living environment	0.948 (0.921, 0.977)	< 0.01	1.021 (0.979, 1.065)	0.34	0.98 (0.952, 1.007)	0.15	0.993 (0.953, 1.034)	0.72	1.101 (0.987, 1.229)	0.09
Quality of life satisfaction	1.002 (0.975, 1.03)	0.88	1.061 (1.016, 1.107)	0.01	0.978 (0.953, 1.004)	0.10	1.029 (0.988, 1.071)	0.16	1.069 (0.965, 1.184)	0.20
Total score of QoL	0.993 (0.983, 1.003)	0.17	1.011 (0.996, 1.026)	0.15	0.989 (0.979, 0.998)	0.02	1 (0.987, 1.014)	0.95	1.017 (0.981, 1.054)	0.36
**Personality characteristics**										
Neuroticism (N)	1.003 (0.994, 1.011)	0.55	0.988 (0.976, 1)	0.06	1.009 (1.001, 1.017)	0.03	0.999 (0.988, 1.011)	0.91	0.984 (0.955, 1.015)	0.31
Extraversion (E)	0.99 (0.981, 1)	0.04	0.985 (0.972, 0.998)	0.02	0.992 (0.983, 1.001)	0.08	0.99 (0.977, 1.003)	0.12	1.022 (0.986, 1.059)	0.24
***Part 2: Relationship of QoL and personality scores with elevated MS components in a sub-group analyses***^**a*#***^										
**13 domains of QoL**										
Companionship	0.991 (0.971, 1.01)	0.345	1.037 (1.001, 1.073)	0.041	0.998 (0.982, 1.015)	0.84	1.019 (0.988, 1.05)	0.231	1.04 (0.967, 1.118)	0.29
Physical activity ability	0.962 (0.944, 0.981)	< 0.01	0.992 (0.965, 1.02)	0.589	0.994 (0.978, 1.011)	0.51	1.011 (0.984, 1.039)	0.429	0.986 (0.93, 1.046)	0.646
Activity opportunity	0.989 (0.968, 1.01)	0.301	1 (0.97, 1.03)	0.989	1.005 (0.986, 1.024)	0.62	1.01 (0.98, 1.041)	0.517	1.102 (1.016, 1.194)	0.018
**Four factors of QoL**										
Living environment	0.934 (0.89, 0.981)	0.006	0.999 (0.931, 1.072)	0.979	0.996 (0.955, 1.039)	0.86	1.028 (0.959, 1.102)	0.437	1.084 (0.924, 1.273)	0.323
**Personality characteristics**										
Extraversion (E)	0.988 (0.972, 1.005)	0.166	0.974 (0.951, 0.998)	0.032	0.992 (0.978, 1.007)	0.31	0.989 (0.966, 1.012)	0.359	1.02 (0.966, 1.077)	0.468

*QoL* quality of life, *MS* metabolic syndrome, *BP* blood pressure, *TGs* triglyceride, *HDL* high-density lipoprotein

^a^Age, sex, region, fatal weight of pregnancy week and mother’s education level were adjusted in GLM model

^#^a sub-group of 951 samples with no MS component in 2014 were included

Subgroup analyses of participants without MS components in 2014 are shown in Table 2. The results showed that PAA (OR (95% CI) 0.962 (0.944, 0.981), *P* < 0.01) and living environment (OR (95% CI) 0.934 (0.89, 0.981), *P* = 0.006) were negatively correlated with central obesity, companionship was positively correlated with elevated BP (OR (95%CI) 1.037 (1.001, 1.073), *P* = 0.04), and activity opportunity was positively correlated with IFG (OR (95%CI) 1.102 (1.016, 1.194), *P* = 0.02). Furthermore, extraversion personality was a protective factor against elevated BP (OR (95% CI) 0.974 (0.951, 0.998), *P* = 0.03).

### Relationship of QoL and personality Taits scores with MS and MS scores in 2019

In the multivariable logistic regression model (Table 3) (adjusted for sex, age, region, foetal weight of pregnancy week and mother’s education level), the results showed that the QoL domain score of PAA was a protective factor against MS (OR (95% CI) 0.966 (0.947, 0.986), *P* < 0.01). In addition, a high extraversion personality score was a protective factor for MS even after adjusting for sex, age, region, foetal weight of pregnancy week and mother’s education level (OR (95% CI) 0.984 (0.968, 1.000), *P* = 0.05). However, the QoL score of the “other” domain (picky eating and surroundings) was positively correlated with MS (OR (95% CI) 1.029 (1.007, 1.052), *P* = 0.01). In the full model, the QoL domain of others (picky-eating and surroundings) was positively correlated with MS (OR (95% CI) 1.048 (1.022, 1.075), *P* < 0.01), and the living environment factor was negatively correlated with MS (OR (95% CI) 0.932 (0.873, 0.994), *P* < 0.05).

**Table 3:** Logistic regression analysis the relationship of QoL and personality traits with adolescent MS

Variables	MS^a^			MS^b^		
	β	OR (95%CI)	*P*	β	OR (95%CI)	*P*
**13 domains of QoL**						
Self-satisfaction	− 0.006	0.994 (0.976, 1.012)	0.51	− 0.006	0.994 (0.975, 1.012)	0.50
Relationship of teacher and pupil	− 0.008	0.992 (0.972, 1.013)	0.45	− 0.008	0.992 (0.971, 1.013)5	0.43
Physical feeling	− 0.006	0.994 (0.976, 1.013)	0.54	− 0.007	0.993 (0.974, 1.013)	0.51
Companionship	− 0.010	0.990 (0.971, 1.009)	0.31	− 0.012	0.988 (0.969, 1.007)	0.23
Parenthood	− 0.003	0.997 (0.978, 1.016)	0.76	− 0.002	0.998 (0.979, 1.018)	0.88
Physical activity ability	− 0.035	0.965 (0.946, 0.985)	< 0.01	− 0.034	0.966 (0.947, 0.986)	< 0.01
Learning ability and attitude	− 0.009	0.991 (0.971, 1.011)	0.38	− 0.010	0.990 (0.969, 1.011)	0.34
Self-esteem	− 0.013	0.987 (0.968, 1.007)	0.20	− 0.014	0.986 (0.966, 1.006)	0.16
Negative emotion	− 0.009	0.991 (0.973, 1.010)	0.35	− 0.009	0.992 (0.973, 1.010)	0.37
Attitude towards doing homework	− 0.015	0.985 (0.964, 1.007)	0.18	− 0.017	0.983 (0.962, 1.005)	0.14
Activity opportunity	− 0.005	0.995 (0.973, 1.017)	0.65	− 0.007	0.993 (0.971, 1.015)	0.53
Living convenience	0.017	1.017 (0.987, 1.048)	0.28	0.014	1.014 (0.984, 1.045)	0.36
Other (picky-eating and surroundings)	0.024	1.024 (1.003, 1.046)	0.03	0.029	1.029 (1.007, 1.052)	0.01
**Four factors of QoL**						
Psychosocial	− 0.014	0.986 (0.967, 1.006)	0.18	− 0.012	0.988 (0.967, 1.009)	0.25
Physical and mental health	− 0.021	0.979 (0.945, 1.014)	0.23	− 0.020	0.980 (0.946, 1.015)	0.26
Living environment	− 0.056	0.946 (0.900, 0.993)	0.03	− 0.050	0.952 (0.904, 1.002)	0.06
Quality of life satisfaction	0.002	1.002 (0.954, 1.052)	0.93	0.008	1.008 (0.959, 1.060)	0.76
Total score of QoL	− 0.014	0.986 (0.970, 1.003)	0.11	− 0.015	0.985 (0.968, 1.002)	0.09
**Personality characteristics**						
Neuroticism (N)	0.008	1.008 (0.994, 1.023)	0.26	0.008	1.008 (0.993, 1.023)	0.28
Psychoticism (P)	0.008	1.008 (0.985, 1.031)	0.51	0.008	1.008 (0.985, 1.032)	0.48
Extraversion (E)	− 0.018	0.982 (0.967, 0.997)	0.02	− 0.016	0.984 (0.968, 1.000)	0.05
**Full model**^**#**^						
Physical activity ability	− 0.012	0.988 (0.966, 1.009)	0.26	− 0.014	0.986 (0.965, 1.008)	0.22
Other (picky-eating and surroundings)	0.045	1.046 (1.02, 1.073)	< 0.01	0.047	1.048 (1.022, 1.075)	< 0. 01
Living environment	− 0.077	0.926 (0.87, 0.986)	0.02	− 0.071	0.932 (0.873, 0.994)	0.03
Extraversion (E)	− 0.012	0.988 (0.971, 1.006)	0.19	− 0.011	0.989 (0.971, 1.007)	0.23

*QoL* quality of life, *MS* metabolic syndrome

^#^The significant variables of QoL or EPQ were entered in the full model

^a^The crude results

^b^Age, sex, region, fatal weight of pregnancy week and mother’s education level were adjusted in each Logistic regression model (dependent variable value: 1 = with MS, 0 = without any one of five MS components), and 1144 samples were included

After adjusting for other covariates, the GLM (Table 4) also revealed that a high PAA score was a protective factor against the MS score (β = − 0.008, *P* < 0.01). In contrast, a high QoL score of the “other” domain was positively correlated with the MS score (β = 0.007, *P* < 0.01). In the full model, other (picky-eating and surroundings) domains were positively correlated with MS even after adjusting for covariates (β = 0.010, *P* < 0.01), and extraversion personality was a protective factor against MS in both the total sample (β = − 0.006, *P* < 0.01) and subgroup (β = − 0.005, *P* = 0.03) analyses after adjustment for covariates.

**Table 4:** GLM analysis the association of QoL and personality traits with adolescent MS score

Variables	MS*			MS**			MS sub-group^#^		
	B	StdErr	*P*	B	StdErr	*P*	B	StdErr	*P*
**13 domains of QoL**									
Self-satisfaction	0.001	0.002	0.73	0.001	0.002	0.82	0.002	0.002	0.33
Relationship of teacher and pupil	− 0.002	0.002	0.38	− 0.001	0.002	0.48	− 0.002	0.003	0.50
Physical feeling	− 0.001	0.002	0.76	− 0.001	0.002	0.65	0.001	0.002	0.96
Companionship	0.001	0.002	0.87	0.001	0.002	0.94	0.002	0.002	0.46
Parenthood	0.001	0.002	0.83	0.001	0.002	0.95	− 0.001	0.002	0.64
Physical activity ability	− 0.008	0.002	< 0.01	− 0.008	0.002	< 0.01	− 0.005	0.002	0.02
Learning ability and attitude	0.001	0.002	0.94	0.001	0.002	0.86	− 0.002	0.003	0.43
Self-esteem	− 0.001	0.002	0.61	− 0.001	0.002	0.51	− 0.003	0.002	0.18
Negative emotion	0.001	0.002	0.58	0.001	0.002	0.67	0.001	0.002	0.97
Attitude towards doing homework	− 0.002	0.002	0.30	− 0.003	0.002	0.15	− 0.001	0.003	0.82
Activity opportunity	0.003	0.002	0.11	0.003	0.002	0.18	0.001	0.003	0.69
Living convenience	0.001	0.003	0.66	0.001	0.003	0.88	− 0.001	0.003	0.77
Other (picky-eating and surroundings)	0.006	0.002	< 0.01	0.007	0.002	< 0.01	0.003	0.003	0.30
**Four factors of QoL**									
Psychosocial	− 0.002	0.002	0.38	− 0.001	0.002	0.66	− 0.001	0.003	0.57
Physical and mental health	− 0.001	0.003	0.68	− 0.001	0.003	0.66	0.001	0.005	0.98
Living environment	− 0.011	0.005	0.02	− 0.007	0.005	0.12	− 0.006	0.006	0.31
Quality of life satisfaction	0.005	0.005	0.28	0.006	0.005	0.19	0.007	0.006	0.25
Total score of QoL	− 0.001	0.002	0.46	− 0.001	0.002	0.40	− 0.001	0.002	0.50
**Personality characteristics**									
Neuroticism (N)	0.001	0.001	0.93	0.001	0.001	0.93	0.001	0.002	0.82
Psychoticism (P)	0.001	0.002	0.99	0.001	0.002	0.78	0.002	0.003	0.57
Extraversion (E)	− 0.004	0.002	0.01	− 0.003	0.002	0.06	− 0.005	0.002	0.03
**Full model**									
Physical activity ability	− 0.001	0.002	0.57	− 0.003	0.002	0.13	0.001	0.003	0.67
Other (picky-eating and surroundings)	0.010	0.003	< 0.01	0.01	0.002	< 0.01	–	–	–
Living environment	− 0.016	0.006	0.01	–	–	–	–	–	–
Extraversion (E)	− 0.005	0.002	< 0.01	− 0.006	0.002	< 0.01	− 0.005	0.002	0.02

*The crude results

**Age, sex, region, fatal weight of pregnancy week and mother’s education level were adjusted in Logistic regression model. QoL: quality of life, MS scores: having one metabolic syndrome components markers one score

^#^Including the samples who have no MS components in 2014 (*n* = 951)

## Discussion

This study is the first prospective cohort study revealing both the longitudinal and cross-sectional correlations between QoL and MS or MS components, as well as the association between personality traits and MS or MS components over an average 12-year follow-up from birth to adolescence in urban–rural areas. We observed that an increased prevalence of MS components and MS, as well as MS score, was associated with QoL score and extraversion personality traits. The QoL domain score of PAA and extraversion personality score were independent protective factors for both MS and MS scores.

Our study revealed that centrally obese children and adolescents were mainly negatively correlated with physical QoL scores, such as PAA and factor score of living environment, and a dose-dependent relationship was found. Our result was consistent with that of other studies [31–33], showing that the obese population had lower health-related QoL (HRQoL) scores, and that weight loss would improve HRQoL [18, 34]. In our study, childhood and adolescent obesity was not significantly correlated with mental aspects of QoL scores, similar to the results of other studies [32]. In addition, this cohort study indicated that the BP level in childhood and adolescents was inversely correlated with the physical QoL score of PAA in adults [35], but it was positively correlated with mental QoL scores and total QoL scores; additionally, the mental QoL score results were not consistent with the results in adults with hypertension [36]. A study by Jing Sun et al. [37] showed that a decrease in BP would improve the physical HRQoL score in adults with hypertension, but this conclusion is controversial.

Research on the relationship between glycolipid indexes and QoL is limited. A study of adult cardiovascular disease illustrated that elevated TG levels were inversely associated with both physical and QoL scores [35]. Our study was the first to reveal that TGs were negatively related to adolescent QoL, and most of the correlations were significant in the health sub group analyses. A previous study [38] of adulthood hypertension showed that elevated HDL-C was positively correlated with the EuroQol five-dimension, three-level (EQ-5D-3L) index and EuroQol visual analogue scale (EQ VAS) score. Accordingly, we found that increased HDL-C was positively correlated with extraversion personality traits in adolescents. Our findings revealed that the QoL score regarding learning ability and attitude has a salient effect on lipid levels in adolescents, but understanding the mechanism requires further research. In addition, FBG and HbA1c levels in type 1 DM were negatively correlated with EQ-VAS (overall health status) [39], but our cohort study with an adolescent population showed that IFG in childhood was only positively correlated with the QoL score of activity opportunity. The controversial results could be explained by the different lifestyles between healthy children and adults with DM, as well as the different QoL questionnaires used between adolescents and adults.

However, a cross-sectional study showed that QoL scores were correlated with an increase in the components of MS, and the physical health domain of QoL had the most significant association [40]. In our study, we found that the PAA domain score and living environment factor score were protective factors against MS and MS scores. Previous studies have shown that health literacy training is a significant way to promote quality of life [41], suggesting that health literacy training should be provided to out in adolescents with low QoL. To our knowledge, this study is the first cohort study with a large sample size that explored the relationship of QoL with MS and MS scores in adolescents.

Personality traits can be associated with MS, but the conclusion remains controversial, and few studies have been conducted in adult populations; no related cohort studies have explored this relationship in adolescents. The results from Japanese adults [20] showed that the E score was positively correlated with TGs, FBG and MS components, and the P score was positively correlated with FBG. However, we found that the E score in adolescents was negatively correlated with MS, central obesity, and SBP and positively correlated with HDL, suggesting that the E score could be age dependent and have a different impact on lipid metabolism between adults and adolescents. A study by Evans [42] reported that extraverted adolescents have less cortisol activity, which is associated with FBG and FI [43]. Therefore, extraversion traits might regulate glycolipid indexes through the hypothalamic–pituitary–adrenal axis pathway [42], which can impact MS by regulating the reactivity of the sympathetic nervous system (SNS) [44], and the SNS has potent effects on insulin secretion and sensitivity [45] and on lipid metabolism [46]. Adolescents with high N scores would be more prone to responding more strongly to a stressor, and our study did not find N to be correlated with MS, inconsistent with the results of a study in adults [47].

### Limitations

Our study has some limitations that should be considered when interpreting the results. First, since this study was a bidirectional cohort study, recall bias could exist for the prenatal variables. We checked the birth certificates to verify the birth weight, stature and gestational age. Second, data on GH and gestational diabetes were collected through a questionnaire, and recall bias existed. However, we collected the same perinatal information in both 2014 and 2019 independently to reduce recall bias and nonresponse. Finally, QoL and personality traits were collected in a cross-sectional manner in 2019, making it difficult to draw conclusions regarding the causality relationships of QoL and personality traits with MS.

## Conclusions

In conclusion, the prevalence of MS was elevated among adolescents. Our findings add to the scarce research evidence regarding the relationships of QoL and personality traits with MS in children and adolescents, which could facilitate the generation of policy suggestions for health management institutes. In this study, we identified the relationships of QoL and personality traits with MS and MS components from both prospective and cross-sectional aspects after adjustment for other covariates. First, the PAA score was inversely correlated with MS and MS scores, suggesting that the establishment of a comprehensive community, family and school intervention model to improve the PAA of children is essential for the control of adolescents with MS. Second, the QoL score of other domains, including picky eating and surroundings, was positively correlated with MS and MS scores, indicating that health education, including literacy training and health dietary habits, should be implemented both at home and in school to improve lifestyles. Finally, the extraversion personality trait was a protective factor against MS and MS score; therefore, primary prevention in childhood health should focus on cultivating children's extraversion personality. Further studies should explore the effect of comprehensive intervention on the control of adolescent MS by improving QoL and personality traits.

## Supplementary Information

**Additional file 1: Figure S1**. The hypothesized framework of this manuscript.

**Additional file 2: Table S1**. Comparation baseline characteristics between included and excluded participants

**Additional file 3: Figure S2**. The structure chart of quality of life

**Additional file 4: Table S2**. Logistic regression analysis of the association of QoL and personality traits with adolescent MS components without adjusting other covariates

## Data Availability

Data are available from Xiaohua Liang (contact via xiaohualiang@hospital.cqmu.edu.cn, or liangxiaohua666@sina.com).

## Abbreviations

MS: Metabolic syndrome
CVDs: Cardiovascular diseases
TGs: Triglycerides
HDL-C: High density lipoprotein cholesterol
SES: Socialeconomic status
QoL: Quality of life
FBG: Fasting blood-glucose
BMI: Body mass index
GH: Gestational hypertension
WC: Waist circumference
BP: Blood pressure
LDL: Low density lipoprotein cholesterol
TC: Total cholesterol
EPQ: Eysenck’s Personality Questionnaire
PAA: Physical activity ability
E: Extraversion
N: Neuroticism
P: Psychoticism
L: Lie scales
IFG: Impaired fasting glucose
LGA: Large for gestational age
SGA: Small for gestational age
IOM: The Institute of Medicine
SNK: Student–Newman–Keuls
GLM: Generalized linear model
SBP: Systolic blood pressure
DBP: Diastolic blood pressure
HRQoL: Health-related QoL
SNS: Sympathetic nervous system
