# Supplementary figures and images for: The association of quality of life and personality characteristics with adolescent metabolic syndrome: a cohort study
Source: Health Qual Life Outcomes. 2021 Jun 8;19:160. doi: 10.1186/s12955-021-01797-7 (PMC8186050; doi:10.1186/s12955-021-01797-7)

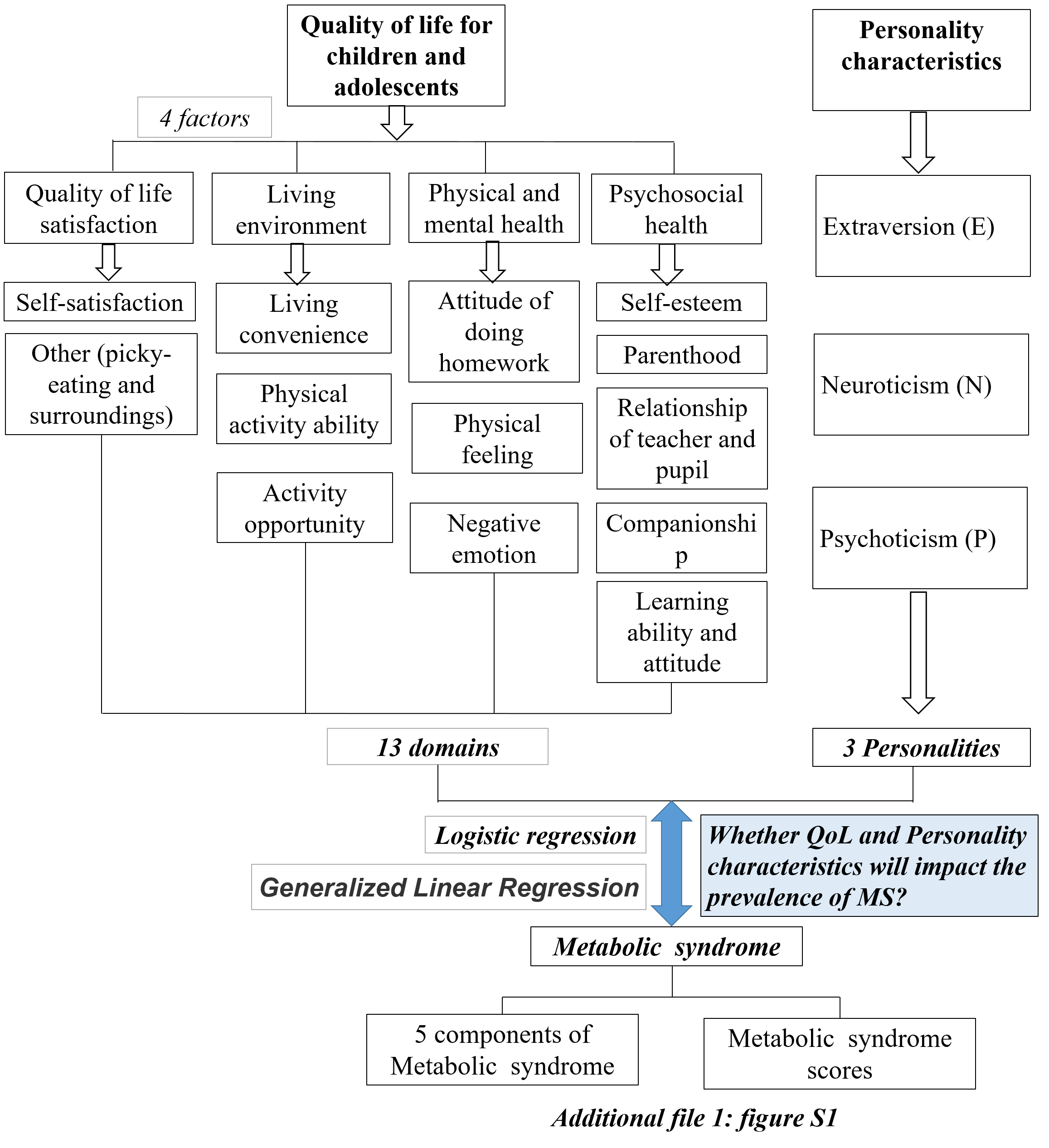

Supplement: Supplementary file 1 — Additional file 1: Figure S1. The hypothesized framework of this manuscript. [file 12955_2021_1797_MOESM1_ESM.tif]

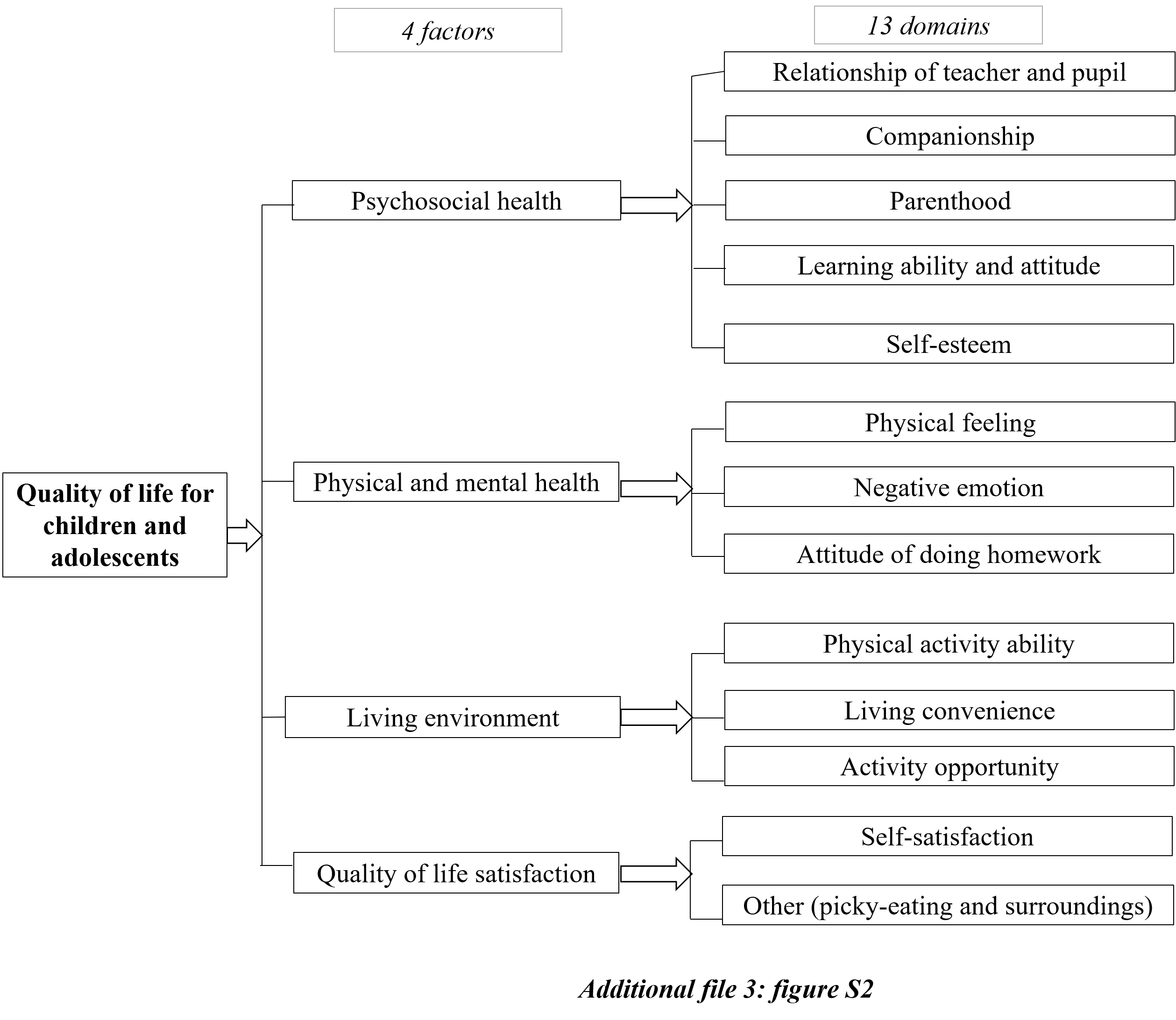

Supplement: Supplementary file 3 — Additional file 3: Figure S2. The structure chart of quality of life [file 12955_2021_1797_MOESM3_ESM.tif]
