# Supplementary material for: The association of quality of life and personality characteristics with adolescent metabolic syndrome: a cohort study
Source: Health Qual Life Outcomes. 2021 Jun 8;19:160. doi: 10.1186/s12955-021-01797-7 (PMC8186050; doi:10.1186/s12955-021-01797-7)
Supplement: Supplementary file 2 — Additional file 2: Table S1. Comparation baseline characteristics between included and excluded participants [file 12955_2021_1797_MOESM2_ESM.docx]

| Table S1. Comparation baseline characteristics between included and excluded participants | | | |
| --- | --- | --- | --- |
| Variables | Included | Excluded | *P* value |
| Age, years | 7.27 (7.24-7.30) | 7.24 (7.20-7.28) | 0.25 |
| Sex, males | 52.16% (980/1879) | 53.18% (494/929) | 0.61 |
| BMI (kg/m^2^) | 16.70 (16.56-16.83) | 16.81 (16.60-17.02) | 0.38 |
| Presented as means with 95% CIs or frequencies | | | |
