# Supplementary material for: The association of quality of life and personality characteristics with adolescent metabolic syndrome: a cohort study
Source: Health Qual Life Outcomes. 2021 Jun 8;19:160. doi: 10.1186/s12955-021-01797-7 (PMC8186050; doi:10.1186/s12955-021-01797-7)
Supplement: Supplementary file 4 — Additional file 4: Table S2. Logistic regression analysis of the association of QoL and personality traits with adolescent MS components without adjusting other covariates [file 12955_2021_1797_MOESM4_ESM.docx]

|  | | | | | | | | | | | | | | | | | | | | |  |
| --- | --- | --- | --- | --- | --- | --- | --- | --- | --- | --- | --- | --- | --- | --- | --- | --- | --- | --- | --- | --- | --- |
| **Table S2. Logistic regression analysis of the association of QoL and personality traits with adolescent MS components** | | | | | | | | | | | | | | | | | | | | |  |
| Variables | Central obesity | | Elevated BP | | |  | | Elevated TGs | | |  | Decreased HDL | | | |  | | Impaired fasting glucose | | |  |
|  | OR (95%CI) | P |  | OR (95%CI) | P | |  | | OR (95%CI) | P | | |  | OR (95%CI) | P | |  | | OR (95%CI) | P | |
| ***Part 1: Relationship of elevated MS components in 2019 with QoL and personality scores in 2019*** | | | | | | | | | |  | | |  |  |  | |  | |  |  | |
| Domains of QoL |  |  |  |  |  | |  | |  |  | | |  |  |  | |  | |  |  | |
| Self-satisfaction | 1.001(0.991,1.01) | <0.01 |  | 1.014(1,1.028) | <0.01 | |  | | 0.994(0.985,1.003) | <0.01 | | |  | 1.005(0.991,1.019) | <0.01 | |  | | 1.011(0.977,1.045) | <0.01 | |
| Physical feeling | 0.998(0.988,1.008) | <0.01 |  | 1.018(1.003,1.033) | <0.01 | |  | | 0.992(0.983,1.001) | <0.01 | | |  | 0.995(0.981,1.009) | <0.01 | |  | | 1.022(0.985,1.061) | <0.01 | |
| Parenthood | 0.999(0.989,1.008) | <0.01 |  | 1.008(0.994,1.021) | <0.01 | |  | | 0.993(0.984,1.002) | <0.01 | | |  | 1.005(0.991,1.019) | <0.01 | |  | | 1.009(0.976,1.043) | <0.01 | |
| Physical activity ability | 0.966(0.956,0.976) | 0.639 |  | 0.990(0.977,1.004) | 0.019 | |  | | 0.992(0.983,1.002) | 0.097 | | |  | 0.997(0.982,1.011) | 0.51 | |  | | 1.015(0.98,1.052) | 0.246 | |
| Learning ability and attitude | 1.000(0.990,1.011) | <0.01 |  | 1.004(0.989,1.018) | <0.01 | |  | | 1.000(0.99,1.01) | <0.01 | | |  | 0.993(0.978,1.008) | <0.01 | |  | | 1.012(0.976,1.049) | <0.01 | |
| Negative emotion | 1.002(0.993,1.012) | <0.01 |  | 1.015(1.002,1.029) | <0.01 | |  | | 0.994(0.985,1.003) | 0.002 | | |  | 1.003(0.989,1.017) | <0.01 | |  | | 1.013(0.98,1.047) | <0.01 | |
| Activity opportunity | 1.003(0.992,1.014) | 0.101 |  | 1.017(1.001,1.034) | <0.01 | |  | | 1.007(0.996,1.018) | 0.006 | | |  | 0.996(0.98,1.012) | <0.01 | |  | | 1.03(0.99,1.071) | <0.01 | |
| Living convenience | 0.998(0.984,1.012) | <0.01 |  | 1.017(0.997,1.039) | 0.174 | |  | | 1.000(0.987,1.013) | 0.102 | | |  | 0.999(0.98,1.019) | 0.653 | |  | | 1.008(0.96,1.058) | 0.395 | |
| Other | 1.015(1.004,1.026) | <0.01 |  | 1.015(1.000,1.030) | <0.01 | |  | | 1.004(0.994,1.014) | <0.01 | | |  | 1.018(1.002,1.034) | <0.01 | |  | | 1.022(0.985,1.06) | <0.01 | |
| **Factors of QoL** |  |  |  |  |  | |  | |  |  | | |  |  |  | |  | |  |  | |
| Psychosocial health factor | 0.997(0.979,1.015) | <0.01 |  | 1.029(1.003,1.056) | <0.01 | |  | | 0.985(0.969,1.002) | <0.01 | | |  | 0.993(0.968,1.018) | <0.01 | |  | | 1.021(0.958,1.088) | <0.01 | |
| Quality of life satisfaction factor | 1.011(0.986,1.036) | <0.01 |  | 1.033(0.997,1.071) | <0.01 | |  | | 0.988(0.966,1.012) | <0.01 | | |  | 1.025(0.989,1.063) | <0.01 | |  | | 1.040(0.953,1.135) | <0.01 | |
| **Total score of QoL** | 0.996(0.988,1.005) | 0.67 |  | 1.009(0.996,1.022) | 0.027 | |  | | 0.994(0.985,1.002) | 0.182 | | |  | 0.998(0.985,1.01) | 0.656 | |  | | 1.008(0.978,1.039) | 0.436 | |
| **Personality Characteristics** |  |  |  |  |  | |  | |  |  | | |  |  |  | |  | |  |  | |
| Neuroticism (N) | 0.999(0.992,1.006) | 0.001 |  | 0.992(0.981,1.002) | <0.01 | |  | | 1.005(0.998,1.012) | 0.017 | | |  | 0.999(0.988,1.009) | <0.01 | |  | | 0.991(0.966,1.016) | 0.001 | |
| Extraversion (E) | 0.995(0.987,1.003) | <0.01 |  | 0.978(0.967,0.988) | <0.01 | |  | | 0.998(0.99,1.006) | <0.01 | | |  | 0.990(0.978,1.001) | <0.01 | |  | | 1.018(0.988,1.05) | <0.01 | |
| ***Part 2: Relationship of QoL and personality scores with elevated MS components in sub-group analyses^*^*** | | | | | | | | | |  | | |  |  |  | |  | |  |  | |
| **13 domains of QoL** |  |  |  |  |  | |  | |  |  | | |  |  |  | |  | |  |  | |
| Self-satisfy | 1.000(0.984,1.017) | <0.01 |  | 1.023(0.996,1.05) | <0.01 | |  | | 0.996(0.982,1.011) | 0.002 | | |  | 1.012(0.986,1.037) | <0.01 | |  | | 1.027(0.976,1.081) | <0.01 | |
| Physical feeling | 0.988(0.971,1.005) | 0.005 |  | 1.013(0.986,1.04) | <0.01 | |  | | 0.996(0.981,1.012) | 0.025 | | |  | 1.002(0.976,1.027) | <0.01 | |  | | 1.035(0.979,1.094) | 0.002 | |
| Parenthood | 0.998(0.982,1.015) | 0.006 |  | 1.009(0.984,1.034) | <0.01 | |  | | 0.99(0.977,1.004) | 0.003 | | |  | 1.013(0.988,1.038) | <0.01 | |  | | 1.016(0.967,1.067) | <0.01 | |
| Learning ability and attitude | 0.99(0.972,1.008) | 0.001 |  | 1.01(0.984,1.037) | <0.01 | |  | | 0.993(0.978,1.009) | 0.001 | | |  | 0.998(0.972,1.025) | <0.01 | |  | | 1.019(0.969,1.072) | 0.001 | |
| Negative emotion | 1.004(0.987,1.021) | <0.01 |  | 1.013(0.989,1.038) | <0.01 | |  | | 0.992(0.978,1.006) | 0.02 | | |  | 1.017(0.992,1.042) | <0.01 | |  | | 1.017(0.971,1.064) | <0.01 | |
| Activity opportunity | 0.994(0.976,1.013) | 0.823 |  | 1.007(0.98,1.036) | 0.006 | |  | | 1.004(0.987,1.02) | 0.002 | | |  | 1.012(0.984,1.041) | <0.01 | |  | | 1.047(0.99,1.108) | 0.001 | |
| Living convenience | 0.989(0.966,1.012) | <0.01 |  | 1.021(0.983,1.06) | 0.111 | |  | | 0.994(0.974,1.015) | 0.65 | | |  | 1.006(0.971,1.043) | 0.748 | |  | | 1.037(0.96,1.121) | 0.888 | |
| Other (picky-eating and surroundings) | 1.001(0.984,1.02) | 0.007 |  | 1.005(0.979,1.031) | <0.01 | |  | | 1.000(0.985,1.016) | 0.016 | | |  | 1.007(0.981,1.034) | <0.01 | |  | | 1.025(0.975,1.077) | <0.01 | |
| ***Four factors of QoL*** |  |  |  |  |  | |  | |  |  | | |  |  |  | |  | |  |  | |
| Physical and mental health | 0.985(0.955,1.016) | 0.001 |  | 1.021(0.975,1.07) | <0.01 | |  | | 0.99(0.963,1.017) | 0.018 | | |  | 1.002(0.957,1.048) | <0.01 | |  | | 1.035(0.943,1.136) | <0.01 | |
| Quality of life satisfaction | 1.004(0.962,1.049) | <0.01 |  | 1.041(0.975,1.112) | <0.01 | |  | | 0.993(0.957,1.03) | 0.005 | | |  | 1.026(0.962,1.093) | <0.01 | |  | | 1.082(0.95,1.232) | <0.01 | |
| ***Total score of QoL*** |  |  |  |  |  | |  | |  |  | | |  |  |  | |  | |  |  | |
| ***Personality Characteristics*** |  |  |  |  |  | |  | |  |  | | |  |  |  | |  | |  |  | |
| Neuroticism (N) | 0.999(0.986,1.012) | 0.054 |  | 0.986(0.967,1.006) | <0.01 | |  | | 1.001(0.99,1.012) | 0.013 | | |  | 1(0.982,1.018) | 0.015 | |  | | 0.98(0.944,1.018) | 0.017 | |
| Extraversion (E) | 0.999(0.985,1.014) | 0.005 |  | 0.965(0.947,0.984) | <0.01 | |  | | 0.998(0.986,1.011) | 0.001 | | |  | 0.986(0.966,1.005) | <0.01 | |  | | 1.019(0.976,1.063) | <0.01 | |
| ^*^a sub-group of 951 samples with no MS component in 2014 were included. QoL: quality of life, MS: metabolic syndrome, BP: blood pressure, TGs: triglyceride, HDL: high-density lipoprotein | | | | | | | | | | | | | | | | | | | | |  |
